# Nutrition and food security impacts of quality seeds of biofortified orange-fleshed sweetpotato: Quasi-experimental evidence from Tanzania

**DOI:** 10.1016/j.worlddev.2019.104646

**Published:** 2019-12

**Authors:** Kelvin Mashisia Shikuku, Julius Juma Okello, Stella Wambugu, Kirimi Sindi, Jan W. Low, Margaret McEwan

**Affiliations:** a Development Economics Group, Wageningen University and Research, 6706 KN Wageningen, Netherlands; b International Potato Center, P.O. Box 22274, Kampala, Uganda; c Food and Agriculture Organization of the United Nations (FAO), Viale delle Terme di Caracalla, 00153, Rome, Italy; d International Potato Center, Kacyiru Road St. 563, Plot No. 1490-Gasabo District, Kigali, Rwanda; e International Potato Center, Box 25171, Nairobi 00603, Kenya

**Keywords:** Orange-fleshed sweetpotato, Biofortified sweetpotato, Virus-free, Nutrition and food security impacts, Quasi-field experiment, Tanzania

## Abstract

This study examined the nutrition and food security impacts of a project that was designed to improve availability of disease-free planting materials of biofortified orange-fleshed sweetpotato (OFSP) in rural Tanzania. Difference-in-difference and matching techniques were employed to estimate causal effects using panel data. Participation in the project increased agronomic and nutritional knowledge of households, raised uptake rate for OFSP varieties, and improved food security status. Effects on nutrition are, however, weak. These results suggest that timely access to quality seeds accompanied by a transfer of skills is important to reduce barriers to adoption of biofortified crops with resulting positive effects on the welfare of rural households. Adequate promotion of both agronomic and nutrition aspects of the technologies may enhance nutrition effects.

## Highlights

• We assessed the impacts of increased availability of disease-free orange-fleshed sweetpotato varieties.
• Availability of orange-fleshed sweetpotato varieties increased awareness, adoption, and food security.
• Investment in seed system alone with less focus on nutrition education will not improve nutrition outcomes.

## 1 Introduction

Malnutrition remains a serious development challenge globally, currently affecting one in three people (Gillespie & van den Bold, 2017). It is a major problem, especially in many countries of sub-Saharan Africa (SSA) where the prevalence of undernourishment and micronutrients deficiency remains high. Estimates from the Food and Agriculture Organization of the United Nations (FAO), for example, showed that the prevalence of chronic undernourishment in SSA rose from 20.8 to 22.7 percent between 2015 and 2016 (FAO, IFAD, UNICEF, WFP, & WHO, 2017). The World Health Organization (WHO) estimates that 59 million children in Africa suffer from some form of malnutrition (WHO, 2017). In addition, the decline in stunting in Africa between 2000 (38.3%) and 2016 (31.2%) was less than one-half the rate of decline in Asia and Latin America. Globally, it is estimated that 250 million children under the age of five years are afflicted with vitamin A deficiency (VAD), one of the major forms of micronutrient related malnutrition in developing countries (WHO, 2017). In SSA, more than 40 percent of the children under five years of age suffer from VAD (Black et al., 2013, Low et al., 2017).

Micronutrient supplementation using high-dose has ordinarily been the most promoted strategy for dealing with VAD. In addition, some food products (such as sugar and oil) have been fortified with vitamin A (Pambo, Otieno, & Okello, 2017). Some studies have indicated that supplementation has not been as effective as predicted (Kenya National Food Fortification Alliance (KNFFA), 2011, Pambo et al., 2014) and fortified processed food products reach urban consumers more than rural. Over the last two decades, complementary efforts to address the high incidence of micronutrient malnutrition in developing countries have promoted food-based approaches including biofortification. Biofortification enriches food staples with key micronutrients, thus using agriculture to solve the micronutrient deficiency and the wider food insecurity problems among vulnerable population (Webb, 2013, Salazar et al., 2016, Low et al., 2017). The strategy, which hinges on the century-old ‘let food be thy medicine’ maxim by Hippocrates, is one of the best known nutrition-sensitive agriculture development strategies.

The appeal of this strategy arises from at least three factors. First, most of the poor households in SSA live in rural areas where agriculture is the main employer (Jayne et al., 2014, Mubila and Yepes, 2017). Second, a large percentage of the food consumed by majority of the rural households comes from own production (Tschirley, Reardon, Dolislager, & Snyder, 2015). Third, incomes in rural populations tend to be low, with a large proportion spent on staple food—leaving very little to spend on other food groups and non-essential household needs.

The biofortification of staple foods has to date focused on resolving the deficiencies of three critical micronutrients, namely zinc, iron and vitamin A. More progress has been made on vitamin A biofortification in SSA compared to the other micronutrients. More than 70 varieties of vitamin A rich orange-fleshed sweetpotato (OFSP) have been bred and released to farm households in SSA since 2009 (Low et al., 2017). Further, it is estimated that more than four million households had received planting materials of improved OFSP varieties by mid-2017. These households have been reached mainly through projects designed to improve nutritional status of targeted households. Projects that promote vitamin A rich foods usually target households with children under five years (especially those 6–23 months old), pregnant women and lactating mothers. These households are the most vulnerable to VAD. Vitamin A deficiency causes impaired growth, risk of morbidity from common infections, and night blindness in children (Birol et al., 2015, Andrade et al., 2017). Most of the projects’ beneficiaries are in Tanzania, Ethiopia, Uganda, Malawi and Mozambique where prevalence of VAD has been stubbornly high. In Mozambique, for instance, more than 69 percent of children below five years of age suffer from VAD (Andrade et al., 2017). In Tanzania, 42 percent of the children are malnourished, with prevalence of VAD estimated to be more than 33 percent among children under five years of age (Tanzania Food and Nutrition Center, 2014).

Early evidence from proof-of-concept studies established that feeding a child on 125 g of vitamin A rich OFSP meets the daily body requirements of vitamin A (Low et al., 2007, Hotz et al., 2012, Low et al., 2017). Amagloh and Coad (2014) demonstrated that OFSP-based complementary food is superior to one based solely on maize. Jones and de Brauw (2015) showed that OFSP, in addition to increasing serum vitamin A levels, also reduces the incidence of childhood diseases such as diarrhea.

Still, empirical research to corroborate the link between agriculture and nutrition is very narrow. Kawarazuka (2010) provided a review of the nutritional impacts of aquaculture and small-scale fisheries. The review showed the different pathways through which aquaculture and small-scale fisheries could improve nutrition status of households in developing countries—including direct consumption of fish, increased incomes, and women participation in such projects. Masset, Haddad, Cornelius, and Isaza-Castro (2012) found, from a review of 23 studies, little support for the hypothesis that agricultural interventions help to reduce under-nutrition. The authors did not interpret the results of their review to imply absence of causal effects, but instead provide useful insights about how factors such as limited statistical power may explain such findings. Hawkes et al., 2012, Webb, 2013 argued that the ‘evidence base’ for the relationship between agriculture and nutrition is poor, except for the community-based research on OFSP.

Lack of sufficient evidence to support the link between agriculture and nutrition has led to the argument that nutrition-sensitive projects often ignore or underestimate the length and complexity of pathways between agriculture to nutrition (Pinstrup-Andersen, 2014). This informs the multiplicity of studies that have focused on unpacking the tenuous link between agriculture and nutrition ((UNICEF, 1990, Van Dorp et al., 2011, Herforth and Harris, 2013, Kadiyala et al., 2014, Kanter et al., 2015, FAO, 2016). Fanzo, 2014, Pingali and Sunder, 2017 further emphasize the multi-sectoral nature of the pathways.

In this study, we use a rich panel dataset collected from an agricultural project conducted in the Lake zone of Tanzania. The project was designed to improve the uptake of sweetpotato, both orange-fleshed and white-fleshed, because the quality of the planting material had been enhanced through removing yield-lowering viruses. The design included an awareness campaign to inform producers of the importance of vitamin A to human health and introduce OFSP as an important source of vitamin. It did not include a community-level nutrition education program, as found in many nutrition-sensitive agricultural interventions (Webb, 2013, Kadiyala et al., 2014, Kanter et al., 2015). Specifically, the study examines: (1) the drivers of participation in the project; (2) the effect of the project on nutrition and agronomic knowledge, and (3) the effects of participation in the project on nutrition and food security outcomes.

The rest of this paper is organized as follows. Section 2 presents the study context and describes the intervention. Section 3 outlines the analytical and empirical methods used. Section 4 explains the data and provides summary statistics. In Section 5, we present and discuss the results while Section 6 concludes and presents the implications of the study for policy and practice.

## 2 Context and intervention

### 2.1 Context

The Lake zone is in the northern part of Tanzania. Sweetpotato (*Ipomoea batatas*) is one of the major crops grown in the zone and across all agro-ecological zones in the country. The crop ranks fourth in importance after maize, cassava, and beans. Farmers primarily grow the crop for home consumption although a few grow it for both home consumption and sale.

Sweetpotato helps to bridge the hunger gap often characterized by an acute shortage of staple foods just before harvest. As a food security crop, the crop can stay in the soil for an extended period making it easy for farmers to ‘store’ it in the field and harvest piecemeal or as needed. The crop can withstand moisture stress caused by lack of or insufficient amounts of rainfall, hence it fills in the food gap during bad weather. Furthermore, sweetpotato does not require much use of external inputs and is often grown without fertilizers and pesticides.

In the Lake zone, a recent increase in the importance of brown streak virus disease affecting cassava is reducing the availability of cassava. Therefore, sweetpotato will become an even more important source of energy for many rural households.

The potential of sweetpotato in the Lake zone is, however, constrained by lack of sufficient and timely access to disease-free planting material (Melka, Namanda, & McEwan, 2011). Two viruses have particularly, been found to be prevalent in the zone. These are the sweetpotato feathery mottle potyvirus (SPFMV) and the whitefly-borne sweetpotato chlorotic stunt virus (SPCSV) (Schaefers and Terry, 1976, Gibson et al., 2000, Adam et al., 2015). Together, the two viruses lead to a severe viral disease called sweetpotato virus disease (SPVD), adversely affecting productivity of most local sweetpotato types (Gibson et al., 2000, Karyeija et al., 2000). Moreover, some of the study areas in the Lake zone experience a prolonged dry period of up to six months. Therefore, for some producers it is a challenge to conserve enough planting materials for the next rainy season. To combat both food and nutrition insecurity in the Lake zone, the International Potato Center (CIP), working with partner organizations, implemented a project aimed at strengthening the seed systems to enhance availability and timely access to virus-free planting materials for both OFSP and non-OFSP varieties.

### 2.2 Intervention

The project, which was named “Marando Bora” meaning “better vines”, was implemented in four regions of the Lake zone, namely Mara, Mwanza, Shinyanga, and Kagera and covered 15 districts. This study, however, focuses on two (Mara and Mwanza) of the four regions and nine districts for which data is available before and after the project. Selection of the two regions and the corresponding districts was purposive because the project’s implementing partners had already been working in the same areas. Marando Bora aimed to use the already existing Great Lakes Cassava Initiative (GLCI) cassava dissemination system, developed by the Catholic Relief Services (CRS) in the lake region, and the voucher system to reach targeted farmers.

The intervention aimed at increasing the availability of quality sweetpotato planting material to farmers at the start of the rainy season in order to promote early planting, enable them to take full advantage of unpredictable rainfall, and to increase root yields (McEwan, Lusheshanija, Shikuku, & Sindi, 2017). Individual farmers and farmer groups were identified to become decentralized vine multipliers (DVMs). The selection criteria for DVMs included previous experience with sweetpotato production, access to adequate land, reliable water for irrigation, ease of access for customers and be reputable in the community. Individual farmers and farmer groups meeting the criteria were identified as DVMs.

Eighty-eight selected DVMs received training and follow-up visits about vine conservation, pest and disease identification and management, agronomic management practices and rapid multiplication techniques (RMTs). The RMTs consisted of using separate multiplication beds, shorter three-node cuttings, closer spacing (10 cm × 20 cm) and more intensive agronomic management. The technology is used to boost the production of vines (for subsequent use as planting material) rather than roots. Multipliers learned how to keep varieties in separate beds, to use labels showing varietal characteristics, and to avoid mixing of different varieties during harvest. Field activities were implemented by the International Potato Center in collaboration with its partners including Catholic Relief Services, Buhemba Rural Agricultural Center (BRAC), Tanzania Home Economics Association (TAHEA), Kituo cha Mafunzo ya Kuboresha Mazingira na Kilimo Adilifu (KIMKUMAKA), and the Rulenge Diocesan Development Office (RUDDO) (Sindi & Wambugu, 2012).

Five varieties (Polista, Ukerewe, Kabode, Ejumula, Jewel) were multiplied and distributed by the DVMs to farmers through a subsidized voucher system. The voucher system was expected to improve access to quality sweetpotato planting material by vulnerable households. Through such a system, farmers were linked with a reliable source of quality planting material where they could also obtain advice and guidance on growing sweetpotato. Vouchers also served to provide a partial guaranteed market for DVMs in order to encourage a more sustainable commercial sweetpotato seed system.

The criteria for identification of voucher beneficiaries were agreed in general village meetings. The criteria included access to land, demonstrated need for planting material, households with children below age five, and women headed households. Women were given priority as sweetpotato is considered “women’s” crop in addition to the significant role played by women on household food security and nutrition. Committees, comprising five to seven members from the local government, Savings and Internal Lending Communities (SILC) and representatives of women’s group leaders knowledgeable about and committed to agriculture development in the area, were formed to help with the listing of the households meeting the criteria, and distributing the vouchers. Voucher beneficiaries were informed to visit DVM sites to collect sweetpotato vines at a date and time that was agreed at sub-village level.

Beneficiary households received 200 cuttings in exchange for the voucher. The total value of the voucher was Tsh 600 (approximately US$ 0.39), with the beneficiary contributing Tsh 100 paid to the DVM in exchange for vines, and the DVM then receiving Tsh 500 after the voucher was redeemed through the Implementing Partner. It is estimated that by the end of 2012, the Marando Bora project had reached 110,000 farmers with quality vines (McEwan et al., 2017). The planting materials, if well conserved, can be used for future planting. In addition to quality vines, sensitization and promotional materials were provided to farmers and other stakeholders to enhance awareness about the benefits of using disease-free vines, nutrition benefits of OFSP varieties, and sources of quality sweetpotato planting materials. Such materials included fact sheets, flyers, posters, pens, and radio scripts, to different project stakeholders^2^.

## 3 Methodology

### 3.1 Analytical framework

**Fig. 1:**
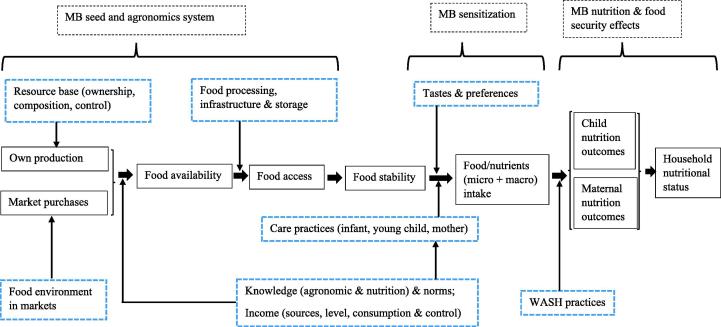
Schematic of analytical framework of this study showing agriculture -nutrition pathways. *Notes:* Adapted from Pinstrup-Andersen and Watson, 2011, Herforth and Harris, 2013, Kadiyala et al., 2014, FAO, 2016. Legend: MB = Marando bora; ---- = MB project interventions and effects; …. = Factors driving changes in the pathway.

The framework of analysis of this paper is based on Fig. 1^3^. It presents our adaptation of the complex agriculture-nutrition pathways based on the current thinking and literature on links between agriculture and nutrition (Herforth and Harris, 2013, Kadiyala et al., 2014, Kanter et al., 2015, FAO, 2016). As shown, a household’s food availability and access, especially in developing countries, importantly depends on own production and market purchases. Households’ ownership of natural resources, the types of resources available (e.g., land, labor, etc), and who has command over the resources play an important role in how much is produced. In the context of this study, land ownership that confers access to lowlands, which enable cross-season conservation of planting materials, can make a positive contribution to how much is produced (Okello, Shikuku, Sindi, & Low, 2015).

Orange-fleshed sweetpotato is a relatively new technology with an underdeveloped market, hence little influence of the market is expected, apart from the effect that higher prices of marketable crops could have on resource allocation away from its production. Similarly, because OFSP is mostly grown for home consumption, access to it will likely not be influenced by transportation and storage services. These services, nonetheless, affect prices, and hence the consumption of other purchased foods. Consumption of purchased foods is expected to affect nutrition outcomes (Webb, 2013).

The lack of or incomplete markets for some of the home-produced foods, especially OFSP, is, however, likely to cause the household to intentionally grow food to meet its nutritional needs (Gillespie & van den Bold, 2017). Development literature has long found that when markets fail to function well, as may be the case for a newly introduced, little-known food crop (such as OFSP), household production and consumption decisions are inseparable (Singh, Squire, & Strauss, 1986). This so-called “non-separability” hypothesis makes agriculture both a direct and indirect influencer of household nutrition. The former is mainly through increased availability of nutrient-rich staple foods to poor households whereas the latter is through incomes earned from agriculture that can be spent on purchasing of nutrition-enhancing goods (including food) and services (Pinstrup-Andersen, 2014, Gillespie and van den Bold, 2017). The income can be derived from farm (e.g., sale of surplus marketable crops and livestock products), off-farm (especially, casual employment) and non-farm activities (such as salaried employment). The latter has effect on household food security (Sraboni et al., 2014, Wagah et al., 2015) as well as the quality of care practices (Rollins et al., 2016), especially where women are involved.

How much of the own-produced OFSP and purchased foods is consumed, hence the micro and macro nutrient intake, not only depends on household income but also on who has command over it. Women’s control over income, and gender equity in general, positively affects food security and household nutrition (Ruel et al., 2013, Chiputwa and Qaim, 2016). Specifically, Malapit, Kadiyala, Quisumbing, Cunningham, and Tyagi (2015) found that women empowerment (and control of production and income) positively affected child dietary diversity, among other nutrition outcomes in Nepal. Van den Broeck and Maertens (2016) found improvements in household food security due to female employment in the Kenyan horticultural industry. Olney, Pedehombga, Ruel, and Dillon (2015) found that ownership and control over productive resources, including income, increased dietary diversity score and nutritional status of women in Burkina Faso.

The effect of agriculture on nutrition is also influenced by nutrition knowledge (Kadiyala et al., 2014) and the knowledge of agronomic practices (Busse et al., 2017). The latter has a direct effect on yields which affects household food supply (availability). The former, which can arise from nutrition education, is associated with improvements in diet quality and care practices. For instance, Olney et al. (2015) found a positive effect of nutrition education, encompassing behavior change communication, on nutrition outcomes in Burkina Faso. Waswa, Jordan, Herrmann, Krawinkel, and Keding (2015) found significant increase in child dietary diversity scores due to knowledge acquired through participation in cooking demonstrations in Kenya.

Previous work introducing OFSP with strong community-level nutrition education components in Mozambique (Low et al., 2007, Hotz et al., 2011) and Uganda (Hotz et al., 2012) demonstrated that such an approach could successfully lead to high uptake of OFSP cultivation, substantially increased intakes of vitamin A rich foods, and reduction in the prevalence of VAD among young children. Findings indicate that a one-year long, group-based community level education program was as effective as a two-year long program (Jones & de Brauw, 2015). In contrast, the Marando Bora project only had a general awareness campaign about the value of consuming OFSP.

Cultural norms also have a strong influence on the intake of micro and macro nutrients. Locks et al. (2015), for instance, discuss the influence local customs have on feeding practices and nutrition. Johnston, Fanzo, and Cogill (2014), on the other hand, find that cultural norms have a strong influence on household food security. The intake of macro and micronutrients is further mediated by tastes and preferences. Indeed, food security literature has food acceptability, or food that ‘meets the needs and preferences’, as one of the four pillars.

The literature also underscores the importance of hygiene and sanitation on nutrition outcomes. OFSP consumption has been associated with increases in serum vitamin A levels and a reduction in hygiene-related childhood diseases such as diarrhea (Jones & de Brauw, 2015). Kang, Suh, Debele, Juon, and Christian (2017) also find positive association between hygiene and child health and nutrition. Fanzo (2014) argues that achieving desired nutrition and public health through food systems need complementary strengthening of water, sanitation and hygiene (WASH) practices.

### 3.2 Empirical approach

The interest in this study lies in estimating causal effects of participation in the ‘Marando Bora’ project. Participation is defined as a dummy variable equal to one if a household received a voucher and obtained vines from a DVM. Within a regression framework, the average treatment effects can, therefore, be estimated according to:

(1) $$y_{\mathrm{it}}=\alpha +\lambda t+\theta W_{i1}t+{\rho W}_{i1}+{\gamma x}_{\mathrm{it}}+\varepsilon_{\mathrm{it}}$$

$$W_{\mathrm{it}}^{*}={\beta z}_{i}+{∊}_{i}$$

(2) $$W_{\mathrm{it}}=\left\{\begin{array}{cc} 1, & if\;W_{\mathrm{it}}^{*}>0\\ 0, & otherwise \end{array}\right)$$

where $y_{\mathrm{it}}$ is the outcome of interest; $W_{\mathrm{it}}^{*}$ is a latent unobserved variable whose counterpart, $W_{\mathrm{it}},$ is observed in dichotomous form only, thus $W_{\mathrm{it}}=1$ represents households that participated in the project and $W_{\mathrm{it}}=0$ represents households that did not participate in the project;t is a time dummy variable; $x_{i}$ is a vector of control variables determining outcome of participating in the project; α and λ capture the individual and time-specific effects, respectively; z is a vector of ‘pre-treatment’ covariates determining participation in the project; β is a vector of parameters capturing the relationships between participation and the pre-treatment covariates; ε and ∊ are error terms of the outcome and participation equations, respectively.

Because households were not randomized into participation and non-participation, participant households might be systematically different from non-participant households. These differences, if they influence outcomes, may invalidate the results from just comparing outcomes between households which participated in the project and those that did not and, possibly, even after adjusting for differences in observed covariates (Imbens & Wooldridge, 2009).

In Eq. (1), the coefficient θ on the interaction between the post-program participation variable $W_{i1}$ and time dummy t gives the average difference-in-differences (DID) effect of households’ participation in the project. The DID estimator requires that the error term $\varepsilon_{\mathrm{it}}$ is uncorrelated with the explanatory variables in the equation, that is, $Cov\left(\varepsilon_{\mathrm{it}},W_{i1}\right)=0;$ $Cov\left(\varepsilon_{\mathrm{it}},t\right)=0;$ $Cov\left(\varepsilon_{\mathrm{it}},x_{\mathrm{it}}\right)=0;$ $Cov\left(\varepsilon_{\mathrm{it}},W_{i1}t\right)=0$ (see also Mendola & Simtowe, 2015). The *parallel-trend* assumption, $Cov\left(\varepsilon_{\mathrm{it}},W_{i1}t\right)=0$, is critical because it implies that unobserved characteristics influencing participation in the project do not vary over time with treatment status.

To control for the possibility of time-invariant selection bias due to initial observables, our empirical estimation combines inverse probability weighting (IPW) and DID methods in a two-stage procedure (Imbens and Wooldridge, 2009, Benin, 2015, Mendola and Simtowe, 2015, Shikuku, 2019). Henceforth, we refer to our approach as IPW-DID. In the first step, $W_{\mathrm{it}}$ is regressed on the vector z using a probit model and propensity score matching technique employed to obtain matched treatment and control observations. The second step involves estimation of Eq. (1) using a DID method based on the matched observations and using the estimated propensity scores as weights. Denote the *weight*s by φ. For farmers in the treatment group, $\varphi =\frac{1}{p}$ whereas for those in the control group, $\varphi =\frac{1}{1-p},$ where p represents estimated propensity scores. Standard errors were clustered at the village level. Clustering of standard errors is important to ensure that standard errors of the estimated treatment effects are not underestimated as this might overestimate the significance of the statistics (Donald & Lang, 2007).

Our estimation relies on an important condition known as unconfoundedness. More specifically, under this assumption, treatment is independent of outcomes once the vector of covariates z is controlled for. The conditional independence assumption does not require the variables in conditioning vector of covariates z and x to be exogenous for the identification of the causal effect of interest (Heckman and Vytlacil, 2005, Diagne and Demont, 2007). The restriction imposed, however, is that values of the variables included in z and x should not change for any farmer when his or her treatment status changes from not-treated to treated (Diagne & Demont, 2007). It is recommended, therefore, that z and x include pretreatment covariates (Heckman and Navarro-Lozano, 2004, Wooldridge, 2005, Diagne and Demont, 2007). In this study, the conditioning set of covariates z and x came from baseline data and that are unlikely to change after ‘treatment’.

The procedure of selecting matched control observations for the treatment observations using the estimated propensity scores improves overlap in the covariate distributions between the treatment and control observations, consistent with the conditional independence assumption (Crump, Hotz, Imbens, & Mitnik, 2006). In line with previous studies, common support was imposed in order to trim observations with propensity scores close to zero or one. Although dropping observations may lead to biased estimates, using the sub-sample can yield higher precision of the estimates than for the overall sample, resulting to greater internal validity at the expense of some of the external validity (Crump et al., 2006)^4^.

Control variables included in Eq. (1) are sex and age of the household head; dependency ratio; literacy; cultivated farm size; access to valley bottom; experience with cassava disease (note that there was an outbreak of cassava brown streak virus during the project period); participation of the household head in casual employment; type of road; value of assets; and regional dummy variable for Mwanza. As a further robustness check, matching was conducted using three different matching algorithms including nearest neighbour, radius, and kernel-based matching.

In order to bolster further confidence in our causal estimates of project participation, we include some placebo tests. In the placebo strategy, baseline nutrition and food security outcomes are regressed on the dummy variable for participation in the Marando Bora project at endline.

## 4 Data, description of variables, and summary statistics

### 4.1 Data

Data were collected through two waves of household surveys. A baseline survey was conducted in September 2010, interviewing 621 households that were selected across the nine districts. The procedure for sampling households for the baseline survey involved three stages. In the first stage, a list of wards participating in the Great Lake Cassava Initiative (GLCI) program was drawn randomly from each of the nine districts. In the second stage, villages were drawn randomly from the selected wards. The third stage involved generating a list of households that were members of either GLCI or Savings and Internal Lending Communities (SILC) in the selected villages, and randomly sampling 621 households for personal interviews (Sindi & Wambugu, 2012).

Between January and March of 2013, a follow up survey was conducted in which 732 households were interviewed. From the 621 households interviewed in the baseline survey, 434 were revisited during the follow up survey. The remaining sample comprised households interviewed at baseline, but not during follow up and those from an addition six districts where the project expanded to in its final year. This study, therefore, used data from the 434 households that were interviewed in both survey rounds^5^. The treatment group comprises a sub-sample (209) of the 621 households interviewed at baseline who actually received and redeemed a voucher for sweetpotato vines from the DVMs at endline. Similarly, the control group comprises households (218) interviewed at baseline, but who did not receive a voucher to redeem for vines from the DVMs at endline. It is important to note that the study design is not a randomized controlled trial (RCT), meaning that households self-selected into receiving a voucher.

During the follow up survey, outcome variables that had been identified for impact assessment measurements were collected. These included the dietary diversity of households and children between six and 24 months of age; production and marketing of sweetpotato and other crops; vines diffusion and access to quality planting materials; farmers’ knowledge about sweetpotato production including diseases, vines conservation, and sweetpotato storage methods; households’ food security status; knowledge about vitamin A; nutritional knowledge and dietary habits; frequency of consumption of vitamin A foods; farmers’ attitudes and perceptions about sweetpotato in general and OFSP in particular; sweetpotato varietal preferences; households’ exposure to shocks; and households’ assets endowment (see Okello et al., 2015).

One relevant caveat should, however, be noted. The baseline and the follow up surveys were conducted in different months. Specifically, the baseline survey was conducted in September 2010 whereas the follow up survey took place in January–March 2013. Although most previous studies use datasets collected in a similar manner as ours, it is possible that results might be different if both surveys are conducted in the same months. Specifically, the findings on nutrition-related outcomes of the project may be influenced by this difference in the baseline and follow up survey periods. Furthermore, there are two planting seasons in most parts of the Lake Zone, namely, November–February and April–July. Thus, household food stocks could have been quite different. In order to address this latter concern, our survey datasets in both survey waves covered the last two completed cropping seasons.

### 4.2 Description and summary statistics of variables

#### 4.2.1 Outcome variables

Our first set of outcome variables measures knowledge and is based on indices constructed from knowledge exams (Table 1). Such exams are recommended as effective ways of measuring knowledge (see for example, Kondylis, Mueller, & Zhu, 2015). Specifically, we construct three continuous knowledge variables: (1) knowledge about sweetpotato production; (2) knowledge about vitamin A foods; and (3) an aggregate knowledge score combining both production and vitamin A foods. We weight each correct answer by the inverse-probability of responding correctly (see also Shikuku, 2019). This approach assigns greater weight to more difficult questions (that is, those to which only a few respondents answered correctly) and lower weights to simpler questions (that is, those to which most respondents answered correctly).

**Table 1:** Summary statistics of outcome variables based on unmatched sample.

Variable	2010			2013		
	All	Treatment	Control	All	Treatment	Control
Production knowledge	16.087	17.427	14.802***	16.095	16.624	15.588
Vitamin A knowledge	3.016	4.081	1.995***	3.023	3.554	2.515***
Combined production and vitamin A knowledge	19.102	21.508	16.797***	19.118	20.178	18.102*
Likelihood to grow OFSP (proportion)	0.033	0.057	0.000***	0.333	0.679	0.000***
Proportion of OFSP	0.051	0.078	0.026***	0.086	0.168	0.007***
Child dietary diversity score	1.412	1.435	1.390	1.696	1.708	1.683
Household dietary diversity score	4.424	4.550	4.303	3.176	3.191	3.161
Food group diversity score	4.756	4.904	4.615	3.316	3.316	3.316
Diversity of vitamin A-rich foods	2.047	2.153	1.945	1.066	1.077	1.055
Child food consumption score	36.149	37.127	35.211	17.856	17.952	17.764
Child frequency of consumption of vitamin A-rich foods (days during past week)	5.096	5.344	4.858	3.557	3.775	3.349
Months of inadequate food provisioning	1.059	1.115	1.005	3.166	3.024	3.303
Consumption of sweetpotatoes at least twice weekly (number of months)	6.419	6.507	6.335	5.356	5.301	5.408
Reliance on relief food (number of months)	0.611	0.627	0.596	0.452	0.311	0.587
Sale of assets (proportion)	0.546	0.531	0.560	0.546	0.502	0.587
Food insecurity score	3.597	3.770	3.431	3.454	3.598	3.317
Revenue from sales of sweetpotatoes (Tsh)	2155.00	1445.00	2835.00	62292.00	59033.50	65416.00
Income from other crops (Tsh)	261518.30	285114.00	238897.00	391770.00	400541.00	383360.40
Woman decides how much to sell and how to use income (proportion)	0.267	0.278	0.257	0.129	0.158	0.101*
Number of Observations	434	212	222	434	212	222

*Notes*: *, *** represent statistical significance difference at 10% and 1% level. OFSP = orange-fleshed sweetpotato.

*Source*: Marando Bora baseline and follow up survey.

Next, we define use of improved sweetpotato varieties. The first variable is a dummy equal to one if a household grew any of the OFSP varieties (Kabode, Jewel or Ejumula) at the time of the follow up survey, and zero if otherwise. The second variable measures the proportion of OFSP roots out of total production of sweetpotato for the household. This second measure specifically aims at capturing the intensity of use of OFSP varieties among the study respondents. The third adoption variable is a dummy equal to one if a household grew a disease-free non-OFSP variety (new Polista) at the time of the follow up survey, and zero if otherwise^6^.

We then construct dietary diversity outcomes^7^. Data on dietary diversity were collected based on 24-hour recall (see Swindale & Bilinsky, 2006). The first outcome is the household dietary diversity score (HDDS) based on eight food groups constituted from 12 categories of food. Specifically, we consider starchy staples, legumes and nuts, milk and dairy products, vegetables, fruits, meat (including organ, flesh and fish meat), eggs, and oil. Each group is a binary variable equal to one if a household member consumed the food during the 24 hours preceding the survey, and zero otherwise. The score is thus a summation across the eight food groups and ranges from zero to eight. The second outcome of dietary diversity is the child dietary diversity score (CHDDS). Similar to HDDS, this indicator is based on eight food groups constituted from the same 12 categories of food. Different from the HDDS, however, we consider organ meat and distinguish between vitamin A-rich and other fruits and vegetables.

The third indicator, which we refer to as “food group diversity” is constructed over ten food groups. Enumerators elicited an inventory of foods consumed in the household with reference to the preceding 24-hour period. Groups were defined as: (1) starchy staples (maize, other cereals, sweet potato, other roots and tubers); (2) legumes and nuts (beans, groundnuts, other pulses); (3) dairy (milk, cheese); (4) organ meats (kidney, liver); (5) eggs; (6) flesh foods (fish, red meat, poultry); (7) vitamin A-rich fruits (mango, papaya, guava); (8) vitamin A-rich vegetables (green leafy vegetables, orange sweet potato); (9) other fruits and vegetables; and (10) oil. The 10 groups were constituted from the 12 categories included in the survey instrument^8^. The food group diversity indicator ranges in value from one to ten. The fourth indicator is a count of the groups containing foods that are sources of vitamin A or beta-carotene, which we call “vitamin A diversity”. Again, we base our construction on the original 12 groups in the survey instrument and the 24-hour period. Food items considered include vitamin A-rich fruits, vitamin A-rich vegetables, eggs, dairy products, red meat, and organ meat. The vitamin A diversity indicator ranges from zero to six.

The fifth indicator we use is the food consumption score (FCS) based on 7-day recall using the protocol developed by Helen Keller International (HKI) (Ramakrishnan, Martorell, Latham, & Abel, 1999). One of the main limitations of the dietary diversity indicators discussed above is that none of them takes into account consumption differences among households within a given food group. Based on the HKI protocol, all food items were grouped into seven specific food groups. All consumption frequencies of food items of the same group were then summed, and values of each group above seven was recoded to seven. For each food group, the value obtained was then multiplied by its weight to create weighted food group scores. A sum of the weighted food groups produced the FCS. Our final indicator of nutrition measured frequency of consumption of vitamin A-rich foods and using a 7-day recall.

Beyond outcomes of dietary diversity, the study further considered food security indicators. The first food security outcome was the months of inadequate household food provisioning (MIHFP) indicator. This indicator captures changes in the household’s ability to address vulnerability in such a way as to ensure that food is available above a minimum level all year round (Bilinsky & Swindale, 2010). Measuring the MIHFP has the advantage of capturing the combined effects of a range of interventions and strategies, such as improved agricultural production, storage and interventions that increase the household’s purchasing power (Bilinsky & Swindale, 2010). To construct the MIHFP score, respondents were probed for the months of the year (in the 12 months preceding the survey) in which the household had less than two meals a day from its own resources (purchases and production). The second indicator measured the number of months in which the household received relief food or food from an external source (in the 12 months preceding the survey). The third indicator asked respondents whether the household had sold an asset in order to have food during a difficult time. The fourth indicator was an aggregate index comprising the different strategies that the household had implemented, during months of acute food shortage, to meet household food needs.

We further consider an outcome measuring income for the sample households. Specifically, we include a dummy variable equal to one if household sold an agricultural or livestock product, and zero otherwise. Finally, we include outcomes of perceptions and practice about OFSP: (1) a dummy equal to one if the household would serve an important visitor with sweetpotato, zero if otherwise; (2) a dummy variable equal to one if the household would consume more sweetpotato if it became richer, zero if otherwise; (3) the number of days in a week when the household consumes sweetpotato.

#### 4.2.2 Explanatory variables

We identify several factors that might affect participation in ‘Marando Bora’ project. Sweetpotato production in SSA has been predominantly a women’s activity (Low, Walker, & Hijmans, 2001). However, there is evidence that men in the household tend to start getting involved in production of women’s crops as they gain value (Dolan, 2001). The effect of sex of the household head on participation in the project is thus ambiguous and an empirical issue. We expect age of the household head to correlate negatively with the probability to participate in Marando Bora project. Older farmers may not be active to search for new knowledge. They may also rely on their accumulated experience in farming and tend to exhibit a higher degree of risk aversion consequently reducing the likelihood to try new technologies.

The effect of dependency ratio on participation is ambiguous. While a higher dependency ratio may encourage households to work hard in order to take care of the dependents, it may also imply reduced availability of labour required to perform farming activities. Farmers may, therefore, be limited in terms of how much time they have to sacrifice off the farm and in search of agricultural information and new technologies. Households with infant babies may, however, find it worthwhile to participate in a project that is promoting interventions aimed at improving the welfare of their babies.

We hypothesize that households which rely on farming for livelihood are likely to invest more in risk-reducing strategies such as virus-free varieties. Similarly, households which have higher literacy rates—measured by the proportion of members who have completed primary education—may participate more in the project. Education may not only create an incentive to actively search for new ideas but also enable farmers to easily understand and implement the knowledge acquired. Furthermore, wealth status of the household may determine whether or not it will participate in the project. We, therefore, include variables on assets ownership, namely size of cultivated land, access to valley bottom, and the monetary value of household assets. Households with a greater endowment of assets may experience reduced liquidity constraints hence increased ability to access improved technologies. Similarly, households with access to a valley bottom may have an enhanced ability to conserve vines during the dry season.

The Lake zone region further tends to experience a high prevalence of cassava disease. It is possible, therefore, that farmers may have an increased willingness to participate in a project seeking to helping in addressing a similar problem although for a different crop – in most cases, cassava farmers also grow sweetpotato. Finally, we control for location characteristics using variables representing type of nearest main road and region fixed effects.

Summary statistics for the explanatory variables for participants and non-participants, at baseline and follow up, are presented in Table 2. Households are predominantly male-headed with an average age of 47 years. Farming is the main source of livelihood for more than 90 percent of the households. About one-quarter of the households engaged in casual employment mainly involving working on other people’s farms. The average household size is eight members with a dependency ratio of about 40 percent. On average, a household has one infant baby. Very few people in a household have completed primary level of education (8%). The average amount of cultivated land is about 2.7 ha. About two-fifths of the sample households have experienced the cassava virus.

**Table 2:** Summary statistics of pre-treatment explanatory variables used in the first-stage probit regression to generate propensity scores.

Variable	Unweighted sample				Weighted sample			
	Control	Treatment	Difference	*t*-statistic	Control	Treatment	Difference	*t*-Statistic
	(1)	(2)	(3)	(4)	(5)	(6)	(7)	(8)
HHH head is male (proportion)	0.820	0.736	0.084	2.106**	0.774	0.795	−0.021	−0.420
Age of the HHH in years	47.829	46.505	1.324	1.165	46.663	47.300	−0.637	−0.440
Main livelihood of HHH is farming (proportion)	0.950	0.948	0.002	0.111	0.929	0.940	−0.011	−0.370
HHH engaged in casual labour (proportion)	0.261	0.240	0.021	0.496	0.234	0.250	−0.016	−0.300
Proportion of HH members with education above primary level	0.078	0.085	0.007	−0.526	0.084	0.081	0.003	0170
Dependency ratio of the HH	0.422	0.387	0.035	2.044**	0.372	0.406	−0.034	−1.650*
Number of infant babies	1.311	1.420	−0.109	−1.098	1.415	1.374	0.042	0.310
Amount of cultivated land (Ha)	2.771	2.661	0.110	0.358	2.547	2.835	−0.288	−0.770
Experience with cassava disease	0.405	0.410	−0.005	−0.105	0.406	0.417	−0.011	−0.180
Access to valley bottom (proportion)	0.662	0.736	−0.074	−1.676*	0.781	0.712	0.070	1.340
Type of nearest main road is earth (proportion)	0.513	0.566	−0.053	−1.097	0.504	0.535	−0.031	−0520
Type of nearest main road is tarmac (proportion)	0.117	0.118	−0.001	−0.026	0.104	0.106	−0.002	−0.060
Value of assets (USD)	28.313	30.872	−2.558	−0.513	31.782	31.162	0.620	0.110
Region is Mwanza (proportion)	0.505	0.722	−0.217	−4.759***	0.708	0.609	0.100	1.750*
Number of observations	222	212			221	208		

*Notes*: *, **, *** represent statistical significance at 10%, 5%, and 1% level. HH means household whereas HHH means household head. 1USD = 1578.5Tsh at the time of the follow up survey.

*Source*: Marando Bora baseline survey

The disaggregation by participation in the Marando Bora project reveals, for the unweighted sample, that participant and non-participant households are similar in most variables at baseline, except for the sex of the household head, dependency ratio, and access to valley bottom. Specifically, 8.4 percent more non-participant than participant households are male-headed whereas dependency ratio is 3.5 percent lower and access to a valley bottom is 7.4 percentage points higher among participant households than non-participant households. Columns (5–8) in Table 2 present descriptive statistics after weighting. As shown, participant and non-participant households are similar after weighting, except in terms of the dependency ratio. But even for the latter variable, the difference is now only statistically different at 10 percent level indicating increased similarity between the participant and non-participant households after weighting. In other words, weighting observations according to the propensity scores reduces difference in average group characteristics.

## 5 Empirical results

### 5.1 Determinants of participation in “Marando Bora” project

Table 3 presents results of the first step probit regression to estimate the propensity scores. As shown a few variables are statistically significantly associated with participation in the project. Specifically, male-headship, age of the household head, and dependency ratio correlated with a reduced likelihood to participate in the project. The average marginal effects of sex and age of the household head and dependency ratio are −0.17, −0.17, and −0.25. Hence, the probability of participation in the project is 17 percentage points lower for male-headed than female-headed households. Similarly, having one more dependent in the household reduced the likelihood of participating in the project by 25 percentage points. The finding relating to gender of the household head suggests that female-headed households are more likely to grow sweetpotato. This could be because sweetpotato does not require many purchased inputs and female-headed households tend to be less endowed financially than male-headed households. The finding of the negative relationship between age of the household head and the probability of participating in the project could be due to differences in the perception of the risk of participating in a new development project.

**Table 3:** Determinants of project participation - first stage probit regression to estimate propensity scores.

Variable	Coefficient	Robust standard error	Marginal effects	*z*-Statistics	*p*-Value
	(1)	(2)	(3)	(4)	(5)
HHH head is male	−0.464	0.166	−0.170***	−2.93	0.003
Age of the HHH in years	−0.457	0.273	−0.168*	−1.70	0.089
Main livelihood of HHH is farming	0.022	0.284	0.008	0.07	0.944
HHH engaged in casual labour	−0.098	0.152	−0.036	−0.64	0.521
Proportion of HH members with education above primary level	0.881	0.548	0.324*	1.67	0.095
Dependency ratio of the HH	−0.691	0.369	−0.254*	−1.94	0.052
Number of infant babies	0.079	0.065	0.029	1.24	0.216
Amount of cultivated land (Ha)	0.015	0.021	0.005	0.63	0.527
Experience with cassava disease	0.082	0.082	0.030	0.64	0.522
Access to valley bottom	0.246	0.246	0.090*	1.78	0.074
Type of nearest main road is earth	0.208	0.208	0.076	1.50	0.134
Type of nearest main road is tarmac	0.102	0.102	0.038	0.50	0.618
Value of assets (natural log)	0.015	0.015	0.005	0.22	0.827
Region is Mwanza	0.640	0.640	0.235***	5.23	0.000
Constant	1.379	1.088	–	–	–
Number of observations	434	434	434	434	434

*Notes*: HH means household whereas HHH means household head. *, *** represent statistical significance at 10% and 1% level. *Source*: Marando Bora baseline survey.

At the same time, we see a positive correlation between participation in the project and literacy rate, as well as access to valley bottoms. The average marginal effects are 0.32 for literacy rate and 0.09 for access to valley bottom. This means that a unit increase in the literacy rate correlates with an increase in the probability of participation in the project by 32 percentage points. Having access to valley bottoms correlates with an increase in the probability of participation by nine percentage points. Households with a higher literacy rate tend to have a greater ability to decode new information and analyze the importance of new technologies (Kassie et al., 2011). Farm households located in Mwanza region are more likely to participate in the project than those in Mara region. These results are in line with the summary statistics and tests presented in Table 2.

### 5.2 Welfare impacts of participation in Marando Bora project

**Fig. 2:**
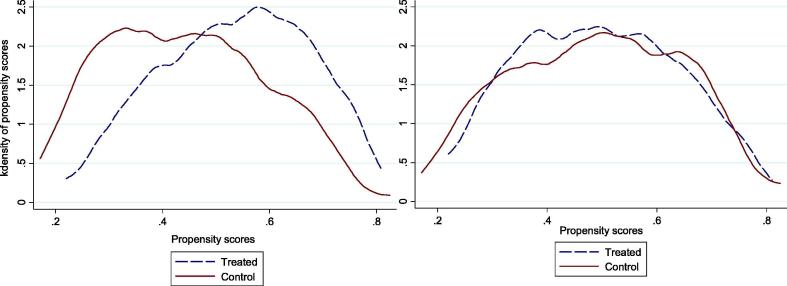
Distribution of propensity scores, without (left panel) and with (right panel) weighting based on Radius matching *Source*: Marando Bora baseline survey.

We now turn to the results of combined difference-in-difference with inverse probability weighting (DID-IPW). Before discussing the causal effects of participation in the project, we first discuss the quality of the matching process. After estimating the propensity scores for the participating and non-participating households we check the common support condition. There is considerable overlap in common support. Among participants, the predicted propensity score ranges from 0.148 to 0.865, with a mean of 0.537, while among non-participants, it ranges from 0.150 to 0.825, with a mean of 0.441. Thus, the common support assumption is satisfied in the region of (0.148, 0.865), with no loss of observations from adopters. This intersection region for the propensity scores is also clear from the common support graph (Fig. 2).

A major objective of the propensity score estimation is to balance the distribution of relevant variables between the households participating and those not participating in the project. Table 4 and Table 5 present results from covariate balancing tests before and after matching. The standardized mean bias for overall covariates used in the propensity score (around 11% before matching) is reduced to about 2.9–9.3 percent after matching. The *p*-values of the likelihood ratio tests indicate that the joint significance of covariates was always rejected after matching, whereas it was never rejected before matching. The pseudo-R^2^ also dropped substantially from 0.07 percent before matching to about 0.004–0.026 percent after matching. Together, the results of the covariates balancing tests indicate that the proposed specification of the propensity score is fairly successful in terms of balancing the distribution of covariates between participating and non-participating households.

**Table 4:** Covariates balancing test results.

Variable	Unmatched sample				Matched sample			
	Control	Treatment	% bias	*t*-Statistic	Control	Treatment	% bias	*t*-Statistic
	(1)	(2)	(3)	(4)	(5)	(6)	(7)	(8)
HHH head is male	0.820	0.736	−20.3	−2.11**	0.763	0.745	−4.2	−0.41
Age of the HHH in years (natural log)	3.836	3.809	−10.7	−1.11	3.803	3.813	3.8	0.38
Main livelihood of HHH is farming	0.950	0.948	−1.1	−0.11	0.926	0.947	9.8	0.90
HHH engaged in casual labour	0.261	0.241	−4.8	−0.50	0.244	0.240	−0.9	−0.09
Proportion of HH members with education above primary level	0.078	0.085	5.0	−0.52	0.085	0.085	0.0	0.00
Dependency ratio of the HH	0.422	0.387	−19.6	−2.04**	0.386	0.390	2.4	0.25
Number of infant babies	1.311	1.420	10.5	1.10	1.413	1.438	2.4	0.24
Amount of cultivated land (Ha)	2.771	2.661	−3.4	−0.36	2.268	2.663	0.8	0.08
Experience with cassava disease	0.405	0.410	1.0	0.11	0.407	0.408	0.3	0.04
Access to valley bottom	0.662	0.736	16.1	1.67	0.744	0.736	−1.9	−0.21
Type of nearest main road is earth	0.514	0.566	10.5	1.10	0.556	0.563	1.3	0.13
Type of nearest main road is tarmac	0.117	0.118	0.3	0.03	0.099	0.120	6.7	0.71
Value of assets	2.798	2.747	−4.7	−0.49	2.791	2.740	−4.6	−0.47
Region is Mwanza	0.505	0.722	45.6	4.75***	0.713	0.721	1.6	0.17
Number of observations	222	212			221	208		

*Notes*: **, *** represent statistical significance at 5% and 1% level. HH means household whereas HHH means household head.

*Source*: Marando Bora baseline and endline surveys.

**Table 5:** Matching quality indicators before and after matching.

Matching algorithm	Pseudo R^2^ before matching	Pseudo R^2^ after matching	LR chi-square (*p*-value) before matching	LR chi-square (*p*-value) after matching	Mean standardized bias before matching	Mean standardized bias after matching
RM	0.072	0.004	43.50*** (0.000)	2.12 (1.000)	11.0	2.9
NNM	0.072	0.022	43.50*** (0.000)	12.11 (0.598)	11.0	8.1
KBM	0.072	0.026	43.50*** (0.000)	14.90 (0.385)	11.0	9.3

*Notes*: RM = Radius matching; NNM = Nearest Neighbour matching; and KBM = Kernel-Based matching.

*** Significant at 1% level.

Table 6 presents project impacts on knowledge: about sweetpotato production (columns 1–2); about vitamin A (columns 3–4); and about both production and vitamin A (columns 5–6). Throughout the paper we present results based on the Radius matching algorithm because it presents the lowest bias. The other matching algorithms provided similar results. We present results for both standardized and unstandardized indices. A standardized index is constructed as the weighted index less its mean and divided by the standard deviation. The results show that participation in Marando Bora project increased knowledge. Two years after baseline, knowledge about sweetpotato production increased by 2.15 score points (column 1) for participating households compared to their non-participating counterparts. This increase in knowledge corresponded to 0.18 and 0.26 standard deviations above the mean (column 2) relative to non-participating households.

**Table 6:** Effect of Marando Bora project on knowledge exposure about sweetpotato production and vitamin A.

	Sweetpotato production		Vitamin A		Production and vitamin A	
Variable	Unstandardized	Standardized	Unstandardized	Standardized	Unstandardized	Standardized
	(1)	(2)	(3)	(4)	(5)	(6)
Post treatment X exposure to Marando Bora project	2.154* (1.083)	0.180* (0.091)	0.844** (0.361)	0.153*** (0.065)	2.998** (1.197)	0.213*** (0.085)
Control variables	Yes	Yes	Yes	Yes	Yes	Yes
R-squared	0.071	0.067	0.027	0.026	0.067	0.064
Number of Observations	840	840	840	840	840	840

*Notes*: In parentheses are robust standard errors clustered at village level. Control variables not reported include sex and age of the household head, whether the main activity of the household head is farming, the proportion of household members engaged in casual employment, the proportion of household members with post-primary education, dependency ratio, number of infant babies, farm size, whether the household experienced cassava disease, access to valley bottom, the floor material of the main house is earth, the nearest main road is tarmacked, assets value (natural log), and regional dummy for Mwanza. *, **, *** represent statistical significance at 10%, 5%, and 1% level.

*Source*: Marando Bora baseline and endline surveys.

Similarly, knowledge score about vitamin A increased by 0.84 (column 3); the corresponding figure in terms of the standardized score was 0.15 (column 4) standard deviations above the mean for the participant households compared to non-participant households. Aggregating across both production and vitamin A, column (5) shows an increase of 3.00 in knowledge score corresponding to 0.21 (column 6) standard deviations above the mean—in terms of the standardized score—for participating households two years after the baseline. These findings indicate that the Marando Bora project had an impact on both agricultural and nutrition knowledge, significantly increasing both outcomes among the project participants. Further, the project increased vitamin A knowledge by a greater magnitude than agricultural knowledge. This finding is in line with *a priori* expectations. Both participants and non-participants were sweetpotato growers, hence already had some knowledge of the agronomic practices. To the contrary, although there had been previous efforts by organizations such as Hellen Keller International to promote OFSP since mid-2000s, use of vitamin A rich sweetpotato, considered among the nutrition knowledge variables, was very low in the study communities and may have contributed to the higher magnitude of nutrition knowledge.

Table 7 shows the effect of the project on the likelihood of growing varieties of OFSP and the proportion of OFSP roots out of total production of sweetpotatoes for the household. The likelihood of growing disease-free non-OFSP varieties also increased substantially. Column (1) shows evidence of high uptake rate of OFSP varieties among participant households two years after the baseline. Specifically, the likelihood of growing OFSP varieties rose by 74 percentage points while that of growing a disease-free non-OFSP variety increased by 60 Percentage points. During the same period, the proportion of OFSP roots out of total production of sweetpotatoes increased by about 16 percentage points (column 2) for participant household compared to non-participant households. These results indicate an increased acceptance of OFSP among households in the lake region of Tanzania and are indicative of early adoption of OFSP by the participant households.

**Table 7:** Effect of Marando Bora project on the likelihood to grow orange-fleshed sweetpotato varieties.

Variable	Likelihood to grow OFSP varieties	Likelihood to grow non-OFSP varieties	Proportion of OFSP roots out of total production
	(1)	(2)	(3)
Post treatment X exposure to project	0.743*** (0.082)	0.602*** (0.050)	0.163*** (0.015)
Control variables	Yes	Yes	Yes
R-squared	0.580	0.538	0.162
Number of Observations	840	840	840

*Notes*: In parentheses are robust standard errors clustered at village level. Control variables not reported include sex and age of the household head, whether the main activity of the household head is farming, the proportion of household members engaged in casual employment, the proportion of household members with post-primary education, dependency ratio, number of infant babies, farm size, whether the household experienced cassava disease, access to valley bottom, the floor material of the main house is earth, the nearest main road is tarmacked, assets value (natural log), and regional dummy for Mwanza. *** represents statistical significance at 1% level.

*Source*: Marando Bora baseline and endline surveys.

In Table 8, we present the impacts of the project on perceptions and practices. The table is intended to assess the likely effects of perceptions and practices of households on preferences for or against sweetpotato. We see that households are willing to consume more sweetpotato even when they become richer. Two years after the baseline, the likelihood to increase consumption of sweetpotato was 13 percentage points (column 2) higher for participant households compared to their counterparts in the non-participant group. This willingness to increase consumption of sweetpotato with wealth is insightful as it perhaps indicates that sweetpotato is not an inferior food in the region. However, this effect may have been driven by change in perception of sweetpotato potentially created by the sensitization activities which emphasized the nutrition benefits, especially of OFSP. The sensitization may have led to sweetpotato being perceived as a more nutritious food. We, however, neither find a statistically significant change in perceptions related to serving an important visitor with sweetpotatoes (column 1) nor number of days that sweetpotato is consumed by a household in a typical week (column 3).

**Table 8:** Effect of participation in Marando Bora project on perceptions and practice about sweetpotatoes.

Variable	Serve SP to visitor always	Consume more SP if richer	Number of days SP is consumed per week
	(1)	(2)	(3)
Post treatment X exposure to project	0.025 (0.045)	0.130*** (0.051)	0.158 (0.243)
Control variables	Yes	Yes	Yes
R-squared	0.092	0.086	0.029
Number of Observations	840	840	840

*Notes*: In parentheses are robust standard errors clustered at village level. Control variables not reported include sex and age of the household head, whether the main activity of the household head is farming, the proportion of household members engaged in casual employment, the proportion of household members with post-primary education, dependency ratio, number of infant babies, farm size, whether the household experienced cassava disease, access to valley bottom, the floor material of the main house is earth, the nearest main road is tarmacked, assets value (natural log), and regional dummy for Mwanza. *** represents statistical significance at 1% level.

*Source*: Marando Bora baseline and endline surveys.

Table 9 presents impacts of the project on nutritional outcomes. Although positive, estimated impacts for household and children dietary diversity scores, food consumption scores, and food group diversity were small in magnitude and not statistically significant. The effect on frequency of consumption of vitamin A foods by children was statistically significant, but only at 10 percent and for matching algorithms that reduced bias the least (that is, Kernel-based and nearest neighbour matching). The finding that the project did not have much influence on nutrition outcome is not surprising, given the project did not have a detailed nutrition education program but instead focused on providing access to better quality vines coupled with information on agronomic practices.

**Table 9:** Effect of Marando Bora project on nutritional outcomes.

Variable	Child DDS	HDDS	FGDS	FGDS vitamin A	FCS	FCS vitamin A
	(1)	(2)	(3)	(4)	(5)	(6)
Post treatment X exposure to project	0.162 (0.183)	0.172 (0.150)	0.138 (0.151)	0.132 (0.096)	1.639 (3.100)	1.482 (1.176)
Control variables	Yes	Yes	Yes	Yes	Yes	Yes
R-squared	0.069	0.143	0.156	0.157	0.072	0.125
Observations	335	840	840	840	335	335

*Notes*: In parentheses are robust standard errors clustered at village level. Child DDS = infant dietary diversity score; HDDS = household dietary diversity score; FGDS = food group diversity score; FCS = food consumption score. Control variables not reported include sex and age of the household head, whether the main activity of the household head is farming, the proportion of household members engaged in casual employment, the proportion of household members with post-primary education, dependency ratio, number of infant babies, farm size, whether the household experienced cassava disease, access to valley bottom, the floor material of the main house is earth, the nearest main road is tarmacked, assets value (natural log), and regional dummy for Mwanza.

*Source*: Marando Bora baseline and endline surveys.

In terms of food security, results in Table 10 show that participation in the project improved the food security status of the households. Although the number of months of inadequate food provisioning score did not improve substantially (column 1), reliance on relief foods significantly decreased by six days (column 2), and the likelihood to sell assets in order to buy foods was 5 percentage points lower (column 3) among participating households compared with non-participating households. However, the latter effect was not statistically significant. Nevertheless, the reduction in the likelihood to sell households assets to bridge food gap may be due to the fact that improved sweetpotato varieties are higher yielding even in poor soils with erratic rains, with some OFSP types having short maturity period (thus providing food much earlier in the season when shortage is most acute), and are grown during two rain seasons of the year in study regions. The finding perhaps suggests the importance of sweetpotato in smoothening consumption. Further, results in column (4) show that participating households engaged in more strategies to improve their food security status compared to non-participating households. This finding may suggest that the project stimulated investment by households in diversified income-generating activities in order to improve food security. Together, the above findings suggest that the project may have increased food stability in the households thus food security.

**Table 10:** Effect of Marando Bora project on food security outcomes.

Variable	Months of inadequate food provisioning	Reliance on relief food	Selling of assets to buy food	Diversification to improve food security
	(1)	(2)	(3)	(4)
Post treatment X exposure to project	−0.038 (0.348)	−0.211** (0.101)	−0.048 (0.053)	0.580** (0.269)
Control variables	Yes	Yes	Yes	Yes
R-squared	0.214	0.098	0.030	0.046
Observations	840	840	840	840

*Notes*: In parentheses are robust standard errors clustered at village level. Control variables not reported include sex and age of the household head, whether the main activity of the household head is farming, the proportion of household members engaged in casual employment, the proportion of household members with post-primary education, dependency ratio, number of infant babies, farm size, whether the household experienced cassava disease, access to valley bottom, the floor material of the main house is earth, the nearest main road is tarmacked, assets value (natural log), and regional dummy for Mwanza. ** represents statistical significance at 5%level.

*Source*: Marando Bora baseline and endline surveys

Income plays an important role in nutrition security of households. Results in Table 11 (columns 1 and 2) show that the likelihood of participating households to sell an agricultural or livestock product rose by 10 percentage points (column 4) two years after the baseline. Results, however, show no significant increase in per capita cash income from sweetpotato sales and crops other than sweetpotato. It is important to note that our variables measure cash income and, therefore, do not value the harvest consumed at home. As such, it is possible that our analysis underestimated impacts of the project on income.

**Table 11:** Effect of Marando Bora project on agricultural and non-agricultural income.

Variable	Sold agricultural or livestock products	Sweetpotato income	Other crop income	Control of income
	(1)	(2)	(3)	(4)
Post treatment X exposure to project	0.0992** (0.0382)	5631.54 (5384.68)	14215.00 (14750.00)	0.0494 (0.0379)
Control variables	Yes	Yes	Yes	Yes
R-squared	0.071	0.080	0.135	0.107
Observations	840	840	840	840

*Notes*: In parentheses are robust standard errors clustered at village level. Control variables not reported include sex and age of the household head, whether the main activity of the household head is farming, the proportion of household members engaged in casual employment, the proportion of household members with post-primary education, dependency ratio, number of infant babies, farm size, whether the household experienced cassava disease, access to valley bottom, the floor material of the main house is earth, the nearest main road is tarmacked, assets value (natural log), and regional dummy for Mwanza. ** represents statistical significance at 5% level. 1USD = 1578.5Tsh at the time of the endline survey.

*Source*: Marando Bora baseline and endline surveys.

One might be concerned that the treated households have different conditions from the control households at the baseline that explain the later improvements in food security. To confirm that households were not chosen to participate in the project because of higher initial likelihood of success, we run a placebo test regressing baseline nutrition and food security outcomes on a dummy participation variable at endline. Results of the placebo tests are presented in Table 12. As shown in Table 12, the coefficients are not statistically significant indicating absence of placebo treatment effects on all nutrition and food security outcomes. The placebo tests therefore cast doubts on the possibility that project participants are better off in terms of nutrition and food security outcomes when they first participate in the project.

**Table 12:** Placebo test on round 1 data to test for differences in baseline.

Variable	HDDS	FGDS	FCS	Months of inadequate food provisioning	Reliance on relief food	Selling of assets to buy food	Food insecurity score
	(1)	(2)	(3)	(4)	(5)	(6)	(7)
Exposure to project	0.249 (0.183)	0.352 (0.214)	3.930 (3.051)	0.209 (0.146)	0.113 (0.089)	−0.045 (0.048)	0.239 (0.309)
Control variables	Yes	Yes	Yes	Yes	Yes	Yes	Yes
R-squared	0.073	0.079	0.134	0.080	0.103	0.053	0.096
Observations	427	427	180	427	427	427	427

*Notes*: In parentheses are robust standard errors clustered at village level. Child DDS = infant dietary diversity score; HDDS = household dietary diversity score; FGDS = food group diversity score; FCS = food consumption score. Control variables not reported include sex and age of the household head, whether the main activity of the household head is farming, the proportion of household members engaged in casual employment, the proportion of household members with post-primary education, dependency ratio, number of infant babies, farm size, whether the household experienced cassava disease, access to valley bottom, the floor material of the main house is earth, the nearest main road is tarmacked, assets value (natural log), and regional dummy for Mwanza.

## 6 Summary, conclusion and study implications

Hidden hunger is a hindrance to economic development in sub-Saharan Africa. Reducing the incidence of vitamin A deficiency (VAD) is a fundamental step towards addressing the problems associated with this form of hidden hunger. One strategy for fighting VAD that has received considerable attention is promotion of biofortified orange-fleshed sweetpotato (OFSP). Consistent with such efforts, a project named “Marando Bora”, meaning “better vines” was implemented by the International Potato Center and its partners in the lake zone region of Tanzania and sought to increase access to quality planting materials of OFSP varieties to rural households. Participant households, therefore, received a bundled intervention comprising increased availability of the quality planting material which were supplied through a subsidized voucher system at decentralized vines multipliers, plus sensitization information about basic agronomic practices and benefits of vitamin A. The objective of this study, therefore, is to assess the nutritional and food security impacts of the bundled intervention. The study adopts an approach that recognizes and attempts to model the long and complex causal pathway between agriculture and nutrition outcomes.

Using a panel dataset, we combine matching techniques with difference-in-differences (DID) methods to estimate causal impacts. Our findings show significant increase in knowledge about sweetpotato production/agronomic practices and of vitamin A, two years after the baseline. More than three-quarters of the participant households retained the planting materials and continued to grow them in subsequent cropping seasons, indicating a very strong early adoption of improved sweetpotato varieties. We further find an increase in the willingness to consume sweetpotato even when households become richer. Participant households experienced a significant reduction in the likelihood to rely on relief food and an increase in options available during times of food shortage, indicating some level of food security. Effects on nutrition outcomes were, however, weak.

To our surprise, our analysis showed that the project increased food security without increasing income and nutrition. While it is not clear the mechanism through which the increase in food security occurred, we speculate that the significant effect of the project on the likelihood to sell agricultural produce, on reduced reliance on relief food, and on increased diversification of strategies employed by households perhaps suggest that the project increased food stability.

Our findings have several implications important for policy. First, our findings clearly imply that the design of nutrition-sensitive projects that concentrate much of the efforts on scaling up the agriculture components (i.e., agronomy), and only a little on nutrition, will have limited effect on nutrition-related outcomes. We have found the nutrition sensitization efforts did influence nutrition knowledge but this influence was not strong enough to effect changes in feeding practices hence dietary diversity and frequency of consumption of vitamin A rich foods. By extension, this implies that building a stronger nutrition component to complement the agricultural component could have resulted in much better nutrition outcomes. Second, the finding that participants had significantly lower reliance on relief food highlights the importance of sweetpotato in filling the food supply gap by helping households meet food needs, and further implies that sweetpotato can relied upon to smooth household food supply over the year. Third, the finding that a significantly large proportion of participants would consume sweetpotato even when they become financially better off implies that sweetpotato is not perceived in study communities as an inferior good. This finding corroborates those of Okello et al. (2015) who also found that such beliefs are unfounded myths. Last, and related to first implication, the study findings imply that investment in sweetpotato seed systems alone without significant or commensurate investment in nutrition education will have weak nutrition outcomes, but will get OFSP introduced into the household and young child diet.

## 7 Contributions

KMS, JJO & SW conceived and planned the paper; KS carried out the data collection; KMS analyzed the data and drafted most sections of the paper; JO & SW drafted some sections and revised the paper; KS contributed some of the paper content; JL critically reviewed and revised the paper; JL, KS & MM conceived and planned the baseline and study.

## Declaration of Competing Interest

The authors declare that they have no known competing financial interests or personal relationships that could have appeared to influence the work reported in this paper.

## Footnotes

2 Because of the bundled nature of the intervention – that is, the intervention included a package of activities (e.g., access to vines and sensitization information) each with possible impact on the outcomes of interest – our design does not seek to isolate the impact of the various components e.g. seed and sensitization information. The estimated effects thus capture combined vines plus the sensitization information.

3 We recognize that international markets play a crucial role in food availability. Although this aspect has not been captured in Fig. 1, we acknowledge its important role in food security.

4 In our specification, only six observations were lost with trimming. The results with and without trimming are similar in terms of the direction of influence and the statistical significance, but the estimates are slightly larger and standard errors are smaller with trimmed sample.

5 The 187 households (HHs) that are excluded from the analysis were not revisited in the follow up survey as their villages were not reached by implementing partners due to logistical reasons, meaning that they were not exposed to the project’s interventions.

6 We include this variable because in addition to OFSP, the project promoted virus-free non-OFSP varieties. Polista is particularly popular in the Lake zone. We thank the anonymous reviewer for the suggestion to include the impact of the project on use of the non-OFSP varieties.

7 Challenges associated with measuring nutrition with anthropometry and testing procedures in developing countries have led to the development of indices of household dietary diversity using cost-effective survey instruments based on recall (Smale, Moursi, & Birol, 2015). Several studies have shown a positive correlation between dietary diversity and improved nutritional parameters: birth weight; child anthropometric status; improved hemoglobin concentrations; caloric and protein adequacy; percentage of protein from animal sources (high quality protein) (e.g. Hoddinott & Yohannes, 2002). Several others validated dietary diversity against nutrient adequacy in developing countries confirming a positive relationship and a consistently positive association between dietary diversity and child growth (Ruel, 2002; Arimond & Ruel, 2002; Working Group on Infant and Young Child Feeding Indicators, 2006; Moursi et al., 2008).

8 The instrument is available upon request.
